# Baicalein Alleviates Osteoarthritis Progression in Mice by Protecting Subchondral Bone and Suppressing Chondrocyte Apoptosis Based on Network Pharmacology

**DOI:** 10.3389/fphar.2021.788392

**Published:** 2022-01-10

**Authors:** Nanxing Yi, Yilin Mi, Xiaotong Xu, Naping Li, Fan Zeng, Ke Yan, Kaiyun Tan, Gaoyan Kuang, Min Lu

**Affiliations:** ^1^ The First Affiliated Hospital of Hunan University of Chinese Medicine, Changsha, China; ^2^ Hunan University of Chinese Medicine, Changsha, China

**Keywords:** osteoarthritis, Baicalein, apoptosis, subchondral bone, inflammation, network pharmacology

## Abstract

As life expectancy increases, Osteoarthritis (OA) is becoming a more frequently seen chronic joint disease. The main characteristics of OA are loss of articular cartilage, subchondral bone sclerosis, and synovial inflammation. Baicalein (Bai), a traditional Chinese medicine extracted from Scutellaria baicalensis Georgi, has been demonstrated to exert notable anti-inflammatory effects in previous studies, suggesting its potential effect in the treatment of OA. In this study, we first predicted the action targets of Bai, mapped target genes related to OA, identified potential anti-OA targets for Bai, performed gene ontology (GO) enrichment, and KEGG signaling pathway analyses of the action targets, and analyzed the molecular docking of key Bai targets. Additionally, the effect and potential mechanism of Bai against OA were verified in mouse knee OA models induced by destabilized medial meniscus (DMM) surgery. GO and KEGG analyses showed that 19 anti-OA targets were mainly involved in the response to oxidative stress, the response to hypoxia and apoptosis, and the PI3K-Akt and p53 signaling pathways. Molecular docking results indicated that BAX, BCL 2, and Caspase 3 enriched in the apoptotic signaling pathway have high binding affinity with Bai. Validation experiments showed that Bai can significantly attenuate the loss of articular cartilage (OARSI score), suppress synovial inflammation (synovitis score), and ameliorate subchondral bone resorption measured by micro-CT. In addition, Bai notably inhibited the expression of apoptosis-related proteins in articular cartilage (BAX, BCL 2, and Caspase 3). By combining network pharmacology with experimental validation, our study identifies and verifies the importance of the apoptotic signaling pathway in the treatment of OA by Bai. Bai may have promising application and potential therapeutic value in OA treatment.

## Introduction

Osteoarthritis (OA) is a severe, debilitating disease that affects the whole joint system and that is characterized by cartilage degeneration, subchondral bone sclerosis, and synovial hypertrophy (Katz et al., 2021). As of 2019, OA is estimated to affect 528 million people worldwide and to be the 15th leading cause of years lived with disability (GBD 2019 Diseases and Injuries Collaborators, 2020). Wage losses due to OA amount to $65 billion, and direct medical costs exceed $100 billion (Hawker, 2019). Persons with knee OA spend an average of approximately $15000 (discounted) over their lifetimes on the direct medical costs of OA (Losina et al., 2015). Despite these staggering statistics, FDA-approved therapies for OA remain limited, and no disease-modifying osteoarthritis drugs (DMOADs) can prevent or restrain the development of OA (Grandi and Bhutani, 2020).

**GRAPHICAL ABSTRACT:**
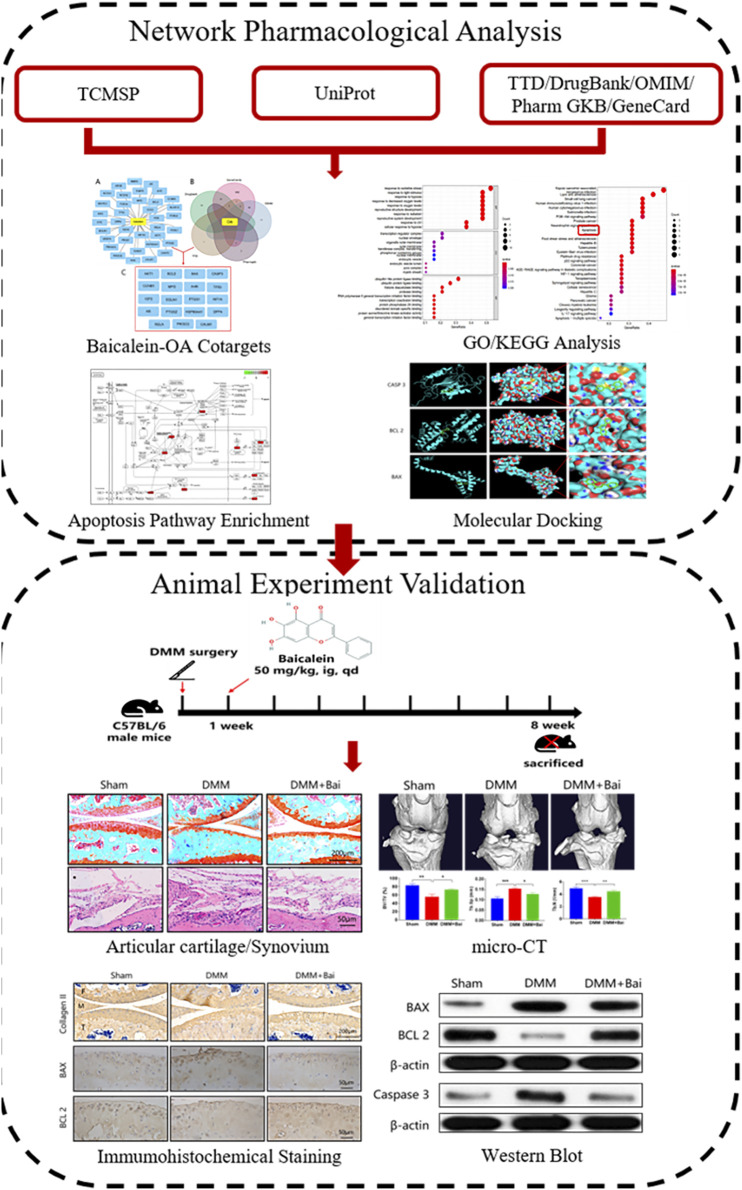


Apoptosis is a highly regulated, active process of cell death involved in development, homeostasis, and aging. Numerous studies have found that chondrocyte apoptosis is positively correlated with the severity of OA (Thomas, et al., 2007; Hwang and Kim, 2015; Li B et al., 2021). Apoptosis has also been speculated to be strongly associated with articular cartilage destruction and matrix degradation in humans (Xue et al., 2019). Therefore, pharmacologic inhibitors of apoptosis may provide a novel treatment option for OA patients.

As a critical component of complementary and alternative medicine, traditional Chinese medicine (TCM) plays an essential role in treating OA. Baicalein (Bai) is a flavonoid extracted from Scutellaria baicalensis Georgi (Huang Qin in Chinese), a medical plant commonly used in different countries for adjuvant therapy for inflammation, diabetes, and cancers (Zhu et al., 2016). Additionally, several studies on the pharmacological mechanism show that Bai has a variety of pharmacological activities, including antioxidant, anti-apoptotic, anti-inflammatory and anti-excitotoxicity effects, and it has a protective effect on mitochondria, and other organelles (Liang et al., 2017). However, little is known about the antiarthritic effects of Bai. Network pharmacology is a new and efficient method to systematically reveal the molecular and pharmacological mechanisms of TCM (Hopkins, 2008). The molecular docking approach is an effective computer modeling technology for docking and analyzing small-molecular weight structures and related disease targets by computer simulation, calculation and analysis of compound biological activity, as well as for screening pharmacodynamic material bases. This method can quickly and efficiently discover new bioactive lead compounds from a database (Ma et al., 2021).

In the present study, we investigated the antiarthritic effects of Bai in knee osteoarthritis (KOA) induced by destabilized medial meniscus (DMM) surgery in mice and explored the underlying molecular mechanisms based on network pharmacology and molecular docking analyses.

## Materials and Methods

### Network Pharmacology-Based Analysis

#### Collection of Bai Putative Targets and OA Targets

The putative targets of Bai were collected from the Traditional Chinese Medicine Database and Analysis Platform (TCMSP) database. The official symbols of Bai targets were generated through the UniProt database (https://www.UniProt.org/) with the species limited to “*Homo sapiens*”. OA-related targets were obtained by searching the database with the keyword “osteoarthritis”. The databases include The Online Mendelian Inheritance in Man (OMIM) (http://omim.org/), the Therapeutic Targets Database (TTD) (http://bidd.nus.edu.sg/group/cjttd/), the PharmGKB (https://www.pharmgkb.org/), the DrugBank (https://www.drugbank.ca/), and the human gene database GeneCards (http://www.genecards.org/).

#### Gene Ontology and KEGG Enrichment Analyses of Co-Targets

To elucidate the role of target proteins that interact with Bai in gene function and signaling pathways, the Database for Annotation, Visualization and Integrated Discovery (DAVID, https://david.ncifcrf.gov/) v6.8 was used to analyze the Gene Ontology (GO) function, and KEGG pathway enrichment of proteins involved in the PPI network. The target proteins involved in the cellular components (CCs), molecular functions (MFs), biological processes (BPs), and pathways were also described.

#### Molecular Docking of Hub Genes and Bai

To test the reliability of Bai-target interactions and explore accurate binding modes, we selected hub genes of key enrichment pathways as molecular receptors and Bai for molecular docking analysis. The raw file of Bai (MOL2 format) was downloaded from the PubChem database (https://pubchem.ncbi.nlm.nih.gov/), Figure 2C raw files download from http://www.rcsb.org/ and after corresponding processing by the AutoDock Tool, the files were finally converted to PDBQT format for molecular docking in PyMOL software (https://pymol.org/2/; version 2.4.1).

### Experimental Validation

#### Animals

Two-month-old male C57BL/6J mice were purchased from Hunan SJA Laboratory Animal Co., Ltd. [Grade SPF, SCXK (Hunan): 2019-0004] and housed under a 12-h light/dark cycle with free access to food and water. After 1 week of adaptive feeding, the mice were subjected to DMM surgery to the right knee or sham surgery as described (Glasson et al., 2007). Briefly, after anaesthetization, a medial articular incision was made to expose the right knee joint. Then, the medial meniscus ligament was transected, and the medial meniscus was gently dissociated. Finally, the medial capsular incision was sutured, and the skin was closed. A sham operation was performed by only opening the joint cavity. All animals included in this study were treated with care that complied with the institutional guidelines established by the Committee of Ethics on Animal Experiments at the Hunan University of Chinese Medicine.

#### Drug Administration

Bai (≥98% purity) was purchased from Yongjian Pharmaceutical Co., Ltd. (Jiangsu, China). Bai was dissolved in 0.5% sodium carboxyl methyl cellulose (5 mg/ml). Eighteen mice were randomly and equally divided into three groups: the sham group, DMM group, and DMM + Bai group. Starting at 1 week after DMM surgery, mice in the Bai group (50 mg/kg) (Sahu et al., 2016; Xiang et al., 2021) or sham DMM group (0.5% CMC-Na) were intragastrically treated once daily. At 8 weeks, all animals were sacrificed, and samples of articular cartilage were harvested.

#### Micro-CT Analysis

The knee joint images of mice were scanned by micro-CT equipment and a reconstruction system (Quantum GX, PerkinElmer). Data were analyzed using data analysis software (CTAn v1.9) and three-dimensional model visualization software (CTVol v2.0). In addition to the visual assessment of structural pictures, quantitative morphometry indices were determined from microtomographic data based on three-dimensional morphometry. A region of interest was identified between the proximal tibia growth plate and tibial plateau. The following indices were subsequently evaluated: bone volume (BV, mm^3^), bone volume fraction (BV/TV, %), trabecular thickness (Tb. Th, mm), trabecular separation (Tb. Sp, mm), and trabecular number (Tb. N, 1/mm).

#### Histological Analysis

Samples of articular cartilage from each group were fixed in 4% paraformaldehyde for 48 h and then decalcified in 10% EDTA (pH 7.4) for 1 month. Next, the cartilage tissues were dehydrated in a graded ethanol series, embedded in paraffin, and cut into 4.0-μm sections. Subsequently, articular cartilage degeneration was evaluated by two staining techniques. Briefly, for hematoxylin and eosin (HE) staining, the cartilage samples were stained with hematoxylin for 5 min and then stained with eosin for 40 s, followed by observation under a microscope. For Safranin O/fast green staining, the samples were stained with 0.02% fast green for 30 min, 1% acetic acid for 10 s, and 1.5% Safranin O for 3 min. After being dehydrated and mounted with neutral balsam, the cartilage samples were observed under a microscope. Two independent experienced researchers who were blinded to the study analyzed cartilage degenerative and synovial changes based on the Osteoarthritis Research Society International (OARSI) scoring system (Pritzker et al., 2006) and synovitis score (Krenn et al., 2006) as previously described.

#### Immunohistochemical Staining

In brief, the paraffin-embedded cartilage tissue sections were dewaxed in xylene and dehydrated in a graded alcohol series, and the sections were subjected to antigen retrieval in proteinase K for 15 min at 37°C. Next, the slices were blocked with 5% BSA in TBST for 30 min, incubated with primary antibody (Collagen II, 1:100, 28459-1-AP, Proteintech; BAX, 1:200, ab32503, Abcam; BCL 2, 1:100, 12789-1-AP, Proteintech) at 4°C overnight, and then incubated with a horseradish peroxidase-conjugated secondary antibody (1:1,000, Beyotime) at room temperature for 2 h. The color reactions were finally performed using a DAB peroxidase substrate (Vector Laboratories) after treatment of the sections with a mixture of avidin and biotinylated horseradish peroxidase. The stained images were photographed using a light microscope and analyzed by Image-Pro Plus software.

### Enzyme-Linked Immunosorbent Assay

Serum levels of the proinflammatory cytokines IL-1β (EK0391, Boster) and TNF-α (FEK0527, Boster) were detected using conventional ELISA kits according to instructions provided by the manufacturer.

#### Quantitative Real-Time PCR

Total RNA was extracted using TRIzol reagent (Takara) according to the manufacturer’s instructions and was reverse-transcribed into cDNA using a PrimeScript RT reagent kit (Takara). Subsequently, real-time PCR was performed using SYBR^®^ GreenER SuperMix (Takara). Relative gene expression was calculated using the 2^−ΔΔCt^ method. The primers used were as follows: β-actin: forward 5′-ACA​TCC​GTA​AAG​ACC​TCT​ATG​CC-3′, reverse 5′-TAC​TCC​TGC​TTG​CTG​ATC​CAC -3′; BAX: forward 5′- TGA​AGA​CAG​GGG​CCT​TTT​TG -3′, reverse 5′- AAT​TCG​CCG​GAG​ACA​CTC​G -3′; Caspase 3: forward 5′- TCT​GAC​TGG​AAA​GCC​GAA​ACT​CT -3′, reverse 5′- AGC​CAT​CTC​CTC​ATC​AGT​CCC​A -3′; BCL 2: forward 5′- TTG​AAA​ACC​GAA​CCA​GGA​ATT​GC -3′, reverse 5′- GTC​CTG​TGC​CAC​TTG​CTC​T -3′.

#### Western Blot

Briefly, cartilage tissues were mixed with RIPA lysate and ground for 10–15 min. Samples were agitated on ice for 30 min, and then the supernatant was collected. Bicinchoninic acid (BCA) analysis was used to qualify the concentration of total proteins. Proteins were then separated by 8% SDS–PAGE and transferred to PVDF membranes (Bio–Rad), followed by blocking with 5% nonfat milk at room temperature for 1 h. After incubating with primary antibody (Caspase 3, 1:2000, 19677-1-AP, Proteintech; BAX, 1:1,000, ab32503, Abcam; BCL 2, 1:1,000, 12789-1-AP, Proteintech, β-actin,1:5,000, 66009-1-Ig, Proteintech) at 4°C overnight, the membranes were washed by TBST and incubated with a secondary antibody at room temperature for 1 h. Later, proteins were scanned and analyzed by a chemiluminescence system and autoradiography.

#### Statistical Analysis

Statistical analysis was performed using GraphPad Prism 7.0 software. All quantitative data are expressed as the mean ± standard deviation (‾χ ± SD). One-way ANOVA followed by Dunnett’s *t*-test was used to determine the significance of differences between groups. A value of *p* < 0.05 was considered statistically significant.

## Results

### Bai Anti-OA by Apoptotic Signaling Pathway

**FIGURE 1:**
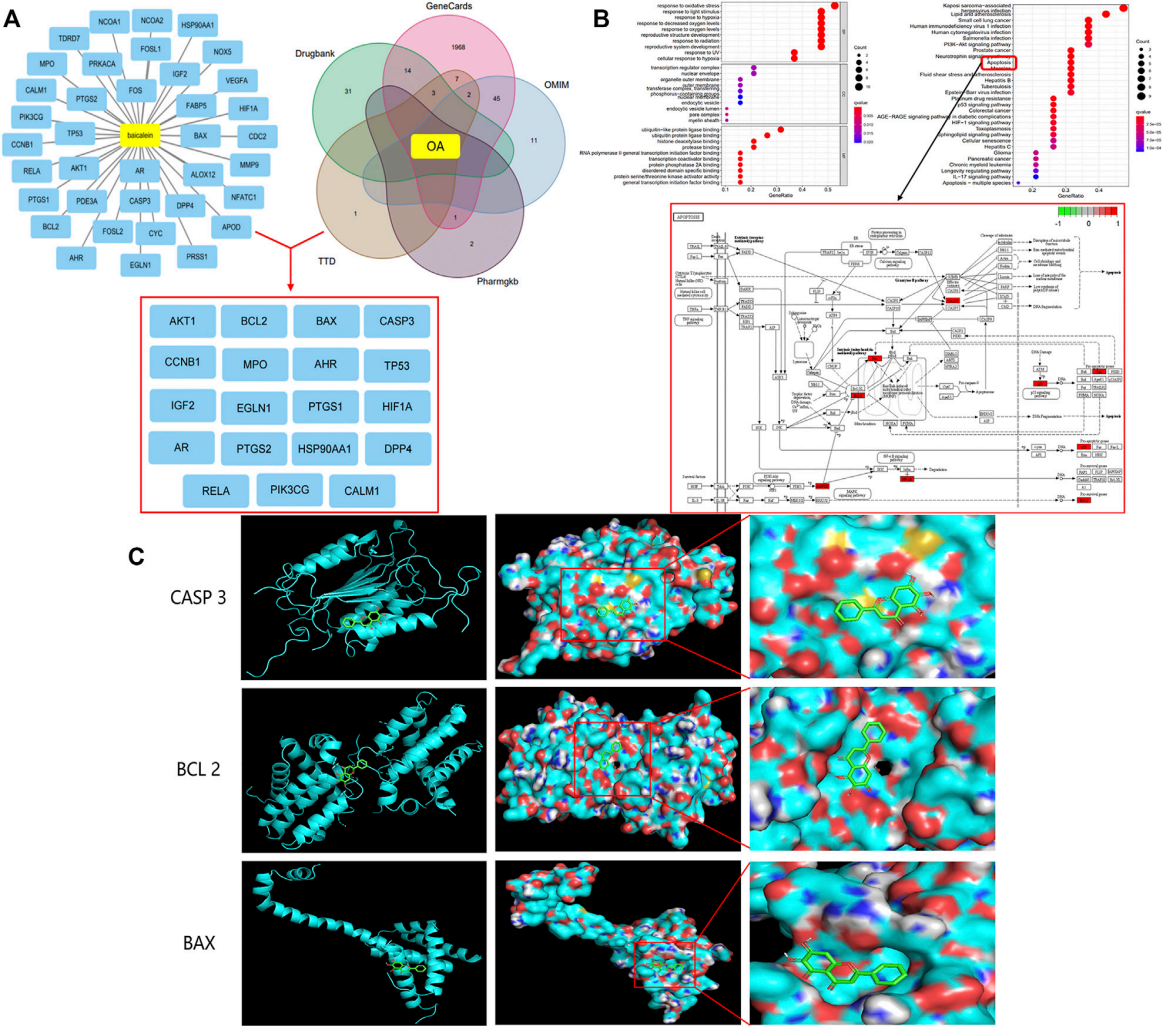
Bai anti-OA by Apoptotic Signaling Pathway. **(A)** Common targets of OA and Bai. **(B)** The top 10 items from the GO analysis for biological process (BP), cellular component (CC), and molecular function (MF). The top 30 pathways of cotargets based on KEGG enrichment analysis and six different targets were enriched in the apoptosis signaling pathway. **(C)** Molecular docking of Bai and BAX, BCL 2, and Caspase 3.

A total of 37 potential targets of Bai were found based on screening in the TCMSP database, 2085 OA-related targets were extracted from the GeneCards, DrugBank, TTD, PharmGKB, and OMIM databases, and a total of 19 anti-OA targets were extracted, including BCL2, CASP3, BAX, and PTGS2 (Figure 1A). In addition, GO enrichment analysis of cotarget proteins was performed using the DAVID database. The top 10 significantly enriched terms in the BP, MF, and CC categories were selected, and the target proteins were mainly involved in the response to oxidative stress and the response to hypoxia. To further clarify the relationship between target proteins and the pathways, we constructed a target–pathway interaction network, and the top 30 pathways were mainly involved in the PI3K-Akt signaling pathway, apoptosis and the p53 signaling pathway. Previous studies have demonstrated that apoptosis plays an important role in the pathogenesis of OA (Aigner et al., 2004; Hwang and Kim, 2015; Gu et al., 2021). Meanwhile, our network results indicate that 6 target proteins are involved in the apoptosis signaling pathway, including BAX, BCL2, and CASP3 (Figure 1B). Finally, molecular docking was carried out to elucidate their binding modes (Figure 1C). The results indicated binding affinity between Bai and 3 targets were lower than −6 (Table 1).

**TABLE 1:** Binding energies of Baicalein and BAX, BCL2, and CASP3.

Molecular	Targets	PDB ID	Affinity (kcal/mol)
Baicalein (C_15_H_10_O_5_)	CASP 3	3DEJ	−6.6
	BCL 2	6O0k	−8.2
	BAX	5W63	−6.8

### Bai Suppresses Synovial Inflammation and IL-1β and TNF-α Production in a DMM-Induced OA Model

**FIGURE 2:**
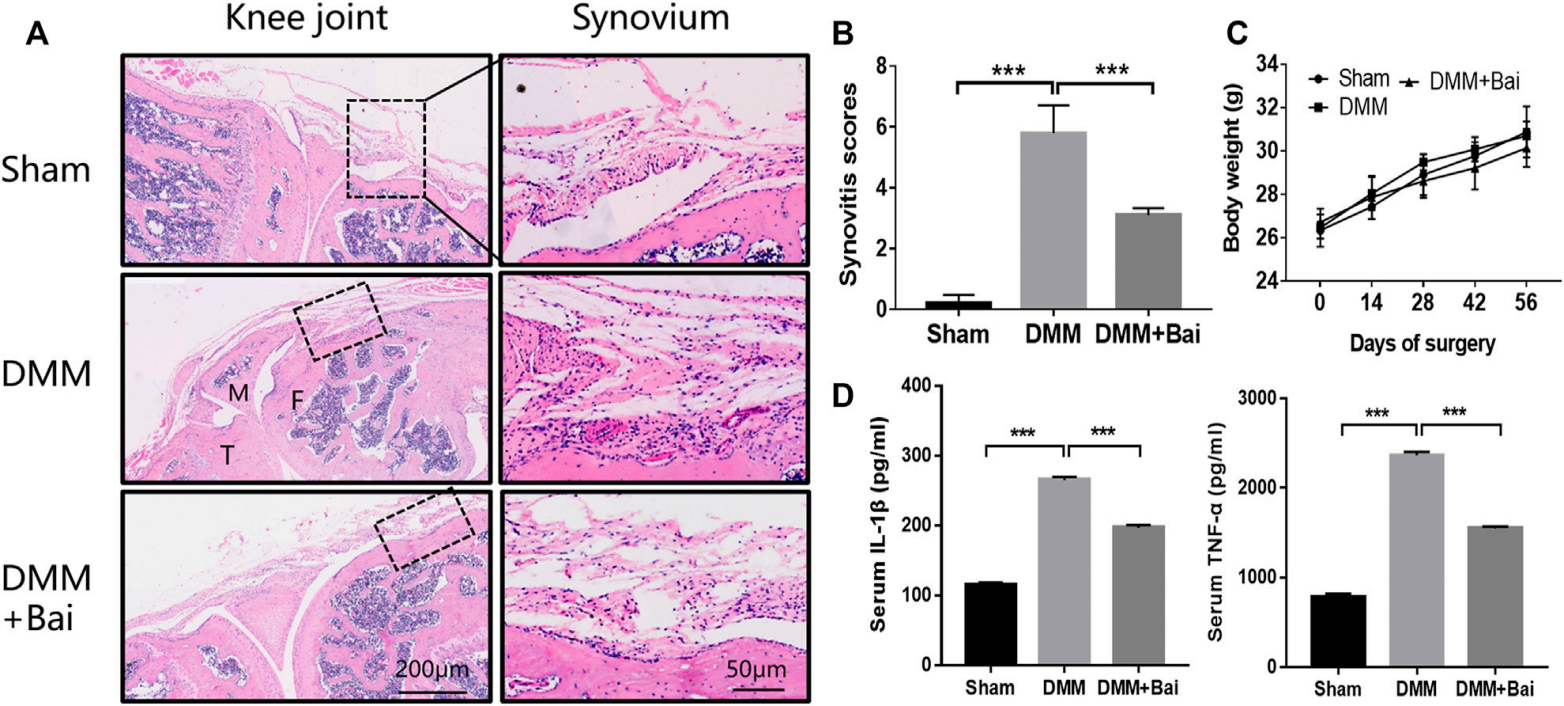
Bai suppresses synovial inflammation and serum IL-1β and TNF-α production in a DMM-induced OA model. **(A)** Hematoxylin and eosin staining of synovial tissue in each group (*n* = 5). **(B)** Synovitis scores in each group at the 8th week postsurgery. **(C)** The body weight in each group over time (*n* = 6). **(D)** IL-1β and TNF-α levels in serum measured by ELISA. Data are shown as the mean ± SD. ***p < 0.001 *vs.* the DMM group.

To investigate the effect of Bai treatment on synovial inflammation in DMM-induced OA, histological analysis with HE staining of the knee joints was performed. Compared with the sham group mice, DMM model mice showed severe synovial hyperplasia, inflammatory cell infiltration into synovial tissues, and pannus formation (Figure 2A). As shown in Figure 2B, Bai significantly attenuated the synovitis scores (Krenn et al., 2006) for the knee joints. Similarly, the levels of the proinflammatory cytokines TNF-α and IL-1β in serum were markedly lower in the Bai-treated group than in the DMM group (Figure 2D).

### Bai Attenuates Cartilage Degradation and Osteophyte Formation

**FIGURE 3:**
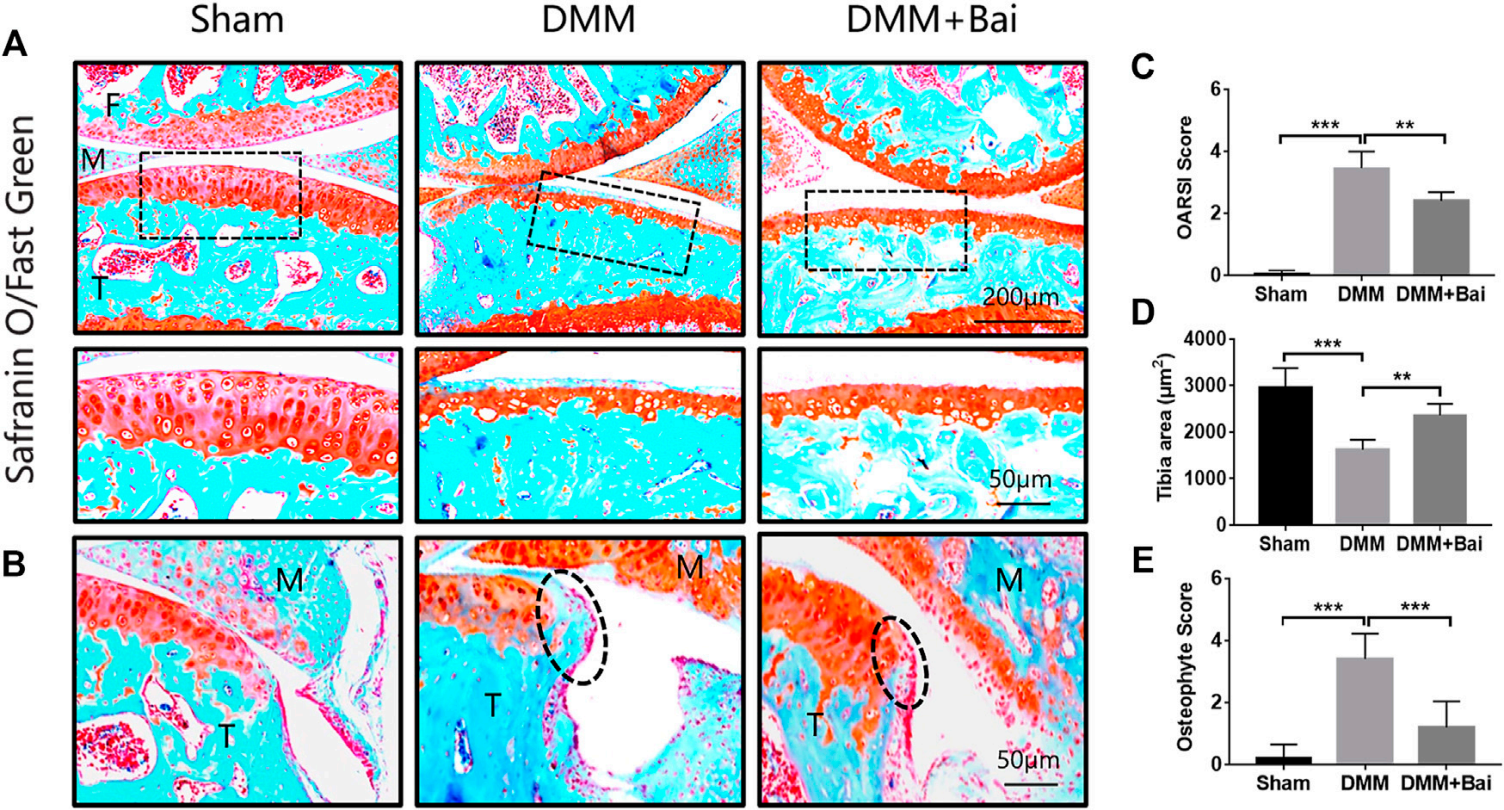
Bai attenuates cartilage degradation and inhibits osteophyte formation. **(A)** Safranin-O/Fast Green staining of articular cartilage in each group. **(B)** Osteophytes are marked by dotted lines. **(C)** OARSI scores for articular cartilage in each group. **(D)** Cartilage area of the tibia. **(E)** Osteophyte scores. Data are shown as the mean ± SD. **p < 0.01, ***p < 0.001 *vs.* the DMM group, *n* = 5. F: Femur, M: Meniscus, T: Tibia.

To assess the effect of Bai treatment on cartilage degradation and osteophyte formation in KOA mice, histological analysis with Safranin O/fast green staining was performed. Articular cartilage exhibited a regular morphological structure in the sham group. Compared with sham group cartilage, DMM group cartilage showed superficial destruction, erosion, proteoglycan loss, and apparent hypocellularity. Compared with the DMM condition, Bai treatment led to a dramatic increase in articular cartilage thickness and amelioration of cartilage damage (Figure 3A). The sections stained with Safranin O/fast green and the cartilage area of the tibia were further assessed with the OARSI histological scoring system (Pritzker et al., 2006). Obviously, the score in the Bai group was significantly lower than that in the DMM group (Figure 3C). Meanwhile, Bai obviously increased the cartilage area of the tibia (Figure 3D). We also found that DMM surgery led to the formation of osteophytes, and treatment with Bai decreased osteophyte scores (Little et al., 2009) in KOA model mice, as shown in Figures 3B,E.

### Bai Ameliorates Subchondral Bone Resorption

**FIGURE 4:**
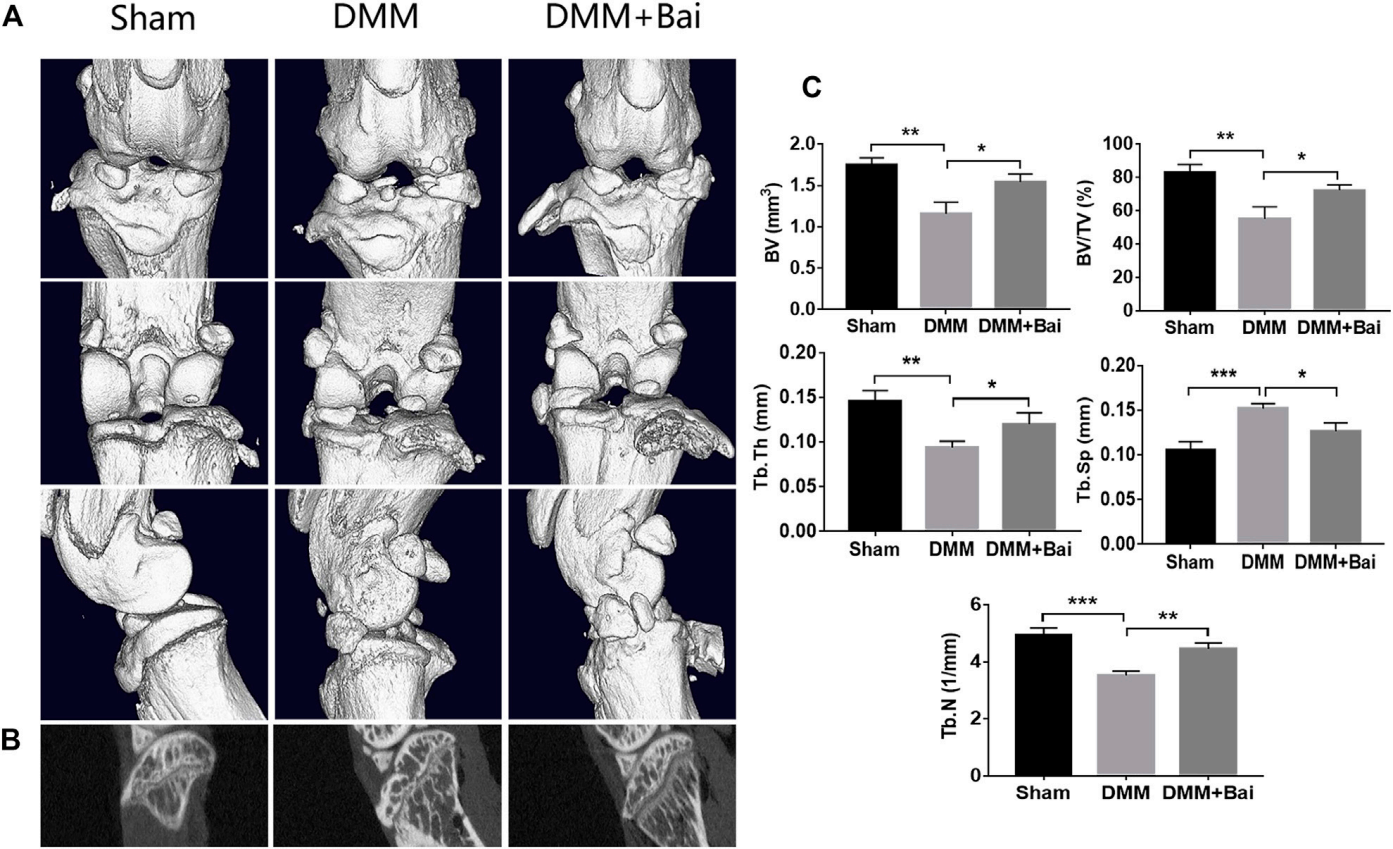
Bai ameliorates subchondral bone resorption. **(A)** Three-dimensional micro-CT images of frontal, posterior and lateral views of the knee joints in each group. **(B)** Sagittal views of medial compartment subchondral bone. **(C)** Quantitative analysis of BV, BV/TV, Tb. Sp, Tb. Th, and Tb.N. Data are shown as the mean ± SD. *p < 0.05, **p < 0.01, ***p < 0.001 *vs.* the DMM group, *n* = 5.

In the early stage of OA, bone loss is associated with increased bone remodeling (Burr and Gallant, 2012). To investigate structural changes in bones in the Bai-treated OA models, three-dimensional imaging was carried out using micro-CT, and quantitative morphometry indices were analyzed. The results indicated that DMM injury induced significant osteophyte formation and bone resorption (Figures 4A,B). Bai significantly increased the bone volume (BV), bone volume fraction (BV/TV), trabecular thickness (Tb. Th), trabecular number (Tb. N), and lowered trabecular separation (Tb. Sp) in the mice after DMM surgery (Figure 4C). The changes in the aforementioned parameters indicated that Bai could suppress subchondral bone remodeling in KOA progression.

### Bai Attenuates Cartilage Degeneration by Inhibiting Chondrocyte Apoptosis

**FIGURE 5:**
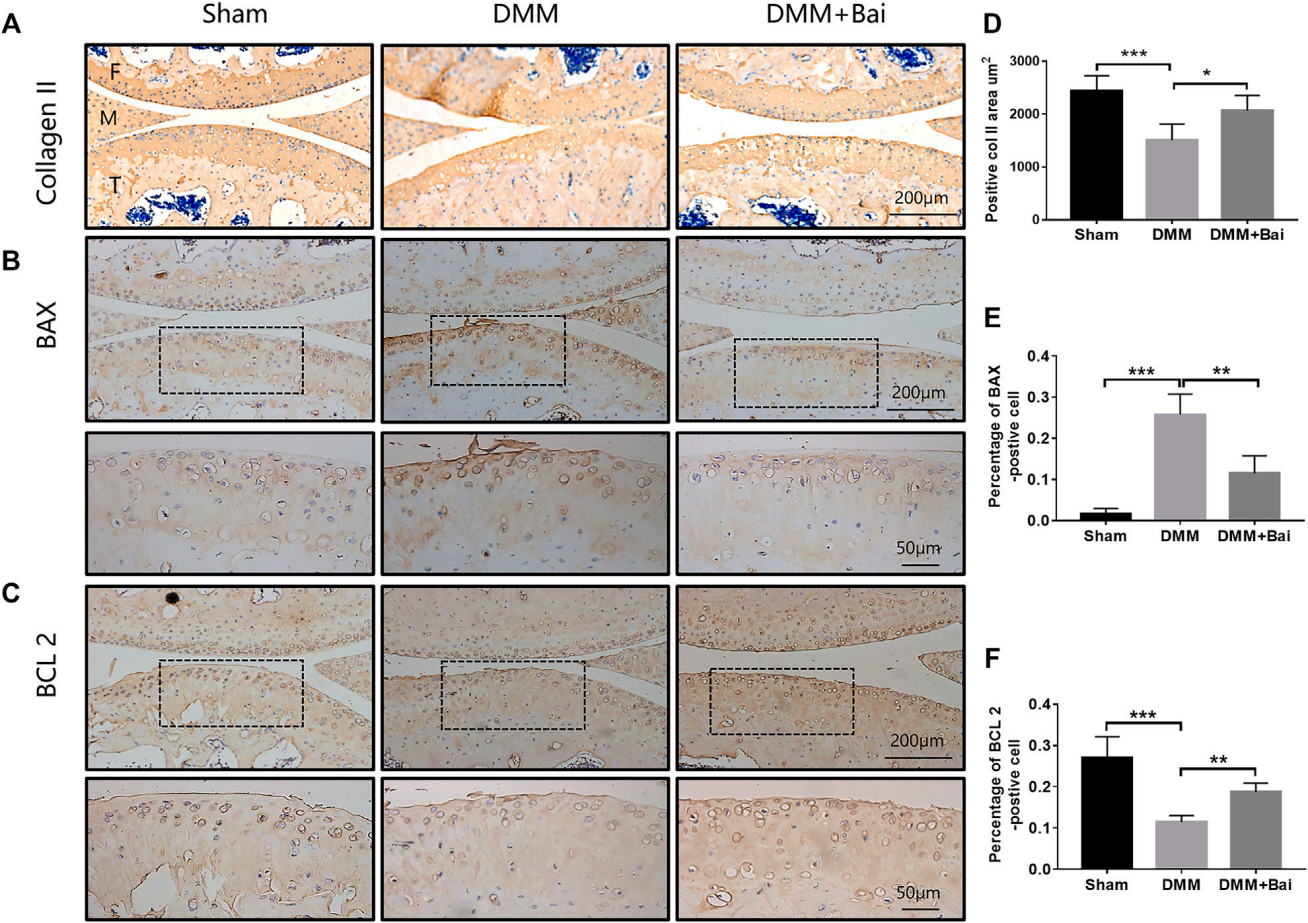
Bai inhibits chondrocyte apoptosis and increases collagen II. **(A–C)** Immunohistochemical staining of collagen II, BAX, and BCL 2 in each group. **(D–F)** Quantitative analysis of collagen II, BAX, and BCL 2. Data are shown as the mean ± SD. *p < 0.05, **p < 0.01, ***p < 0.001 *vs.* the DMM group, *n* = 5. F: Femur, M: Meniscus, T: Tibia.

Chondrocyte apoptosis is an important cause of articular cartilage degeneration (Yu et al., 2021). We further investigated the expression of collagen II, BAX, and BCL 2 by immunohistochemical staining. As shown in Figures 5A,D, Bai treatment increased collagen II expression and markedly improved the positive collagen II area in articular cartilage compared with the DMM condition. At the same time, the percentage of BAX-positive DMM-treated cells was inhibited by Bai (Figures 5B,E). The percentage of BCL 2-positive cells was increased after Bai treatment (Figures 5C,F).

### Bai Regulates mRNA and Protein Expression of Apoptosis-Related Genes in KOA Mice

**FIGURE 6:**
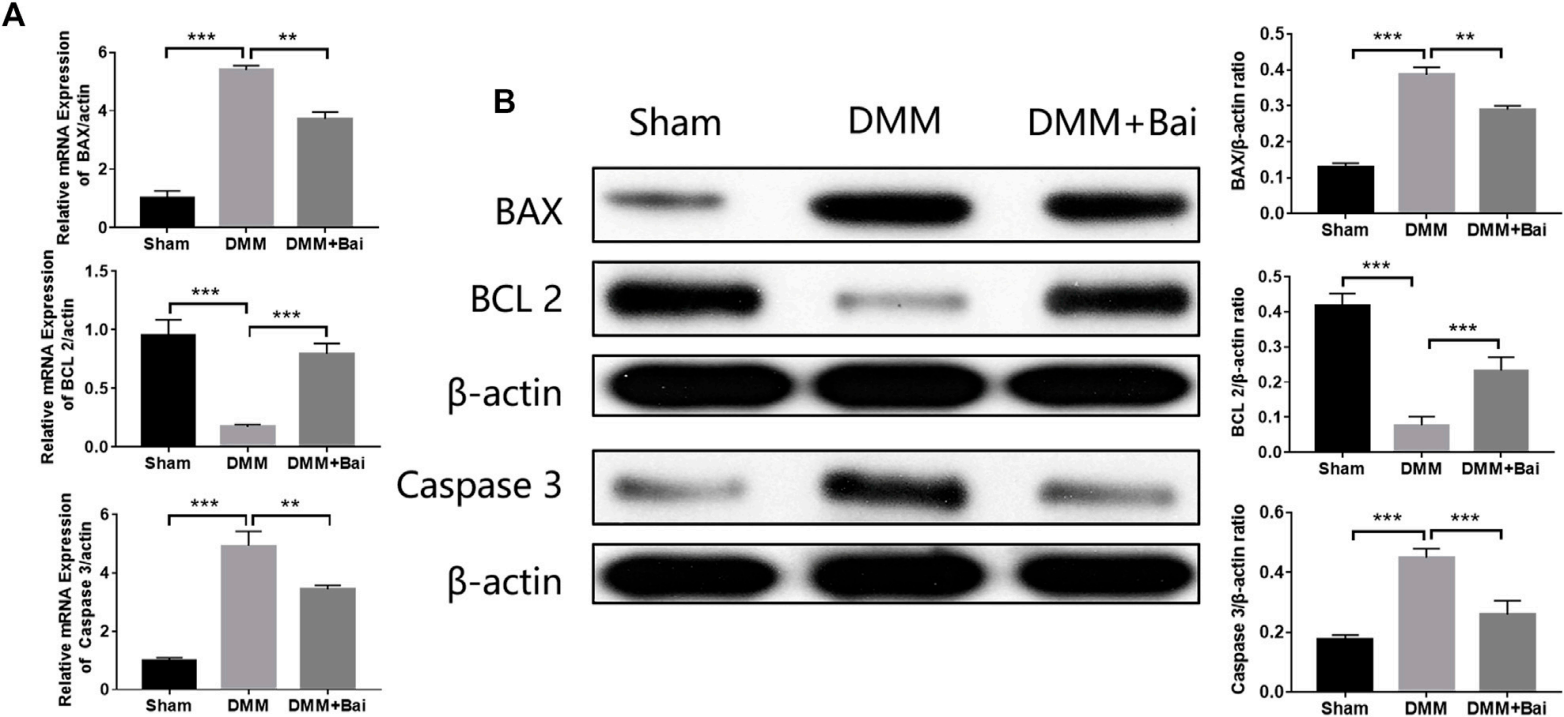
Bai attenuates the mRNA and protein levels of BAX, BCL 2, and Caspase 3. **(A)** mRNA levels of BAX, BCL 2, and Caspase 3. **(B)** Protein levels of BAX, BCL 2, and Caspase 3 were measured by Western blotting. Data are shown as the mean ± SD. **p < 0.01, ***p < 0.001 *vs.* the DMM group.

To verify the mechanism of Bai in treating OA by inhibiting chondrocyte apoptosis based on molecular docking, the expression levels of several key proteins (BAX, BCL 2, Caspase 3) involved in apoptosis signaling pathways were detected. As shown in Figure 6A, the mRNA levels of BAX and Caspase 3 in chondrocytes were enhanced after DMM surgery, but the mRNA levels of BCL 2 in chondrocytes were decreased. However, Bai treatment effectively regulated the mRNA levels of these apoptosis-related molecules. High expression levels of BAX and Caspase 3 are typical indicators of cell apoptosis and can promote cartilage degeneration to OA (Musumeci et al., 2015). These indicators were markedly increased after DMM surgery, and Bai notably attenuated the protein expression of BAX and Caspase 3, while BCL 2 protein showed the opposite trend (Figure 6B).

## Discussion

OA is typically characterized by degeneration and loss of articular cartilage and involves all tissues of the joint, including subchondral bone, and the synovial membrane (Hawker, 2019). Although some progress has been achieved in determining the pathogenesis of and therapeutic options for OA, this disease remains a major obstacle to human health and has high morbidity. A previous study showed that Bai exerted anti-osteoarthritic properties by reducing MMP activities and expression in chondrocytes (Chen et al., 2015). In this study, our data demonstrate that Bai alleviates the OA progression in mice by inhibiting articular chondrocyte apoptosis based on network pharmacological analysis and molecular docking and plays a role in reducing cartilage destruction, alleviating synovial inflammation, and increasing subchondral bone remodeling. To our knowledge, this is the first study to utilize network pharmacology in combination with animal experiments to identify the key role of the apoptotic signaling pathway in the therapeutic effects of Bai against OA. In addition, our results indicate that a network pharmacology approach is a powerful tool to identify molecular targets of herbal formulations and ingredients.

Bai is a natural product with anti-inflammatory properties (Ren et al., 2021). The top targets and signaling pathways of Bai’s anti-OA effect were identified by network analysis, and BAX, BCL2, and Caspase 3 were enriched in apoptotic signaling pathways. Moreover, molecular docking showed that Bai has high binding affinity for the top targets (BAX, BCL2, and Caspase 3). Accumulating studies have revealed that articular chondrocyte apoptosis occurs under the action of endogenous or exogenous stimulation in patients with OA (Qiu et al., 2021; Sun et al., 2021). Chondrocyte apoptosis is one of the important reasons for injury to and loss of articular cartilage and an important mechanism for the occurrence and development of OA (Wang et al., 2021). Our immunohistochemical staining and western blot results further demonstrate that Bai reduces the mRNA and protein expression of the apoptosis-related molecules BAX, BCL 2, and Caspase 3 in chondrocytes, implying that Bai inhibits chondrocyte apoptosis to alleviate cartilage injury and delay OA progression.

Through ELISA, we found that Bai significantly inhibited the expression of TNF-α and IL1β. Studies have shown that the cytokines IL-1β and TNF-α can promote inflammation and apoptosis of chondrocytes, inhibit the expression of aggrecan, and stimulate the expression of MMPs, thus contributing to the pathogenesis of OA (Wang and He, 2018; Wu et al., 2019). Therefore, inhibiting the expression of the inflammation-related genes IL-1β and TNF-α is beneficial for OA remission. In addition, synovial inflammation is one of the main pathological changes in OA, which induces angiogenesis and causes inflammatory cytokines in the serum to accumulate in the joint synovium, leading to aggravated synovial inflammation (Li W et al., 2021). In this experiment, the results demonstrate that Bai inhibits the formation of small blood vessels in the synovial membrane, reduces inflammatory cell infiltration, and significantly decreases the inflammation score of the synovial membrane.

Increasing evidence shows that osteoclast activity is increased in early OA, disturbing the equilibrium between bone formation and resorption, which can ultimately lead to a marked reduction in subchondral bone thickness (Marijnissen et al., 2002). Furthermore, the reduction in the thickness of the subchondral plate was associated with densification of the subchondral plate and cartilage loss (Bellido et al., 2010). In the late stage, OA is characterized by decreased bone resorption and the development of subchondral sclerosis (Chen et al., 2018). In our study, micro-CT scanning results showed that Bai is beneficial for increasing the bone volume fraction of trabecular bone and the number of trabecular bones and decreasing the separation of trabecular bone. Therefore, Bai slows the OA process by reducing the loss of subchondral bone and improving the bone microstructure.

## Conclusion

In conclusion, the current study shows that Bai plays a therapeutic role in OA by inhibiting chondrocyte apoptosis, alleviating cartilage destruction, reducing synovial inflammation, and improving subchondral bone microstructure. Our results also indicate that a network pharmacology approach is a powerful tool for exploring the molecular targets of herbs. Accordingly, our study suggests that Bai has potential as a drug to slow the pathological progression of OA. Of course, a limitation of our study is that we did not investigate the direct mechanism of the apoptotic pathway affecting OA pathogenesis *in vitro*, and further research is needed in the future.

## Data Availability

The datasets presented in this study can be found in online repositories. The names of the repository/repositories and accession number(s) can be found in the article/Supplementary Material.
