# Supplementary material for: Baicalein Alleviates Osteoarthritis Progression in Mice by Protecting Subchondral Bone and Suppressing Chondrocyte Apoptosis Based on Network Pharmacology
Source: Front Pharmacol. 2022 Jan 10;12:788392. doi: 10.3389/fphar.2021.788392 (PMC8784526; doi:10.3389/fphar.2021.788392)
Supplement: Supplementary file 1 [file DataSheet1.ZIP › Additional files/Additional files statement.docx]

Additional files statement

Dear Editors,

Exclude the Figure legend, Additional files are uploaded for review purpose only.

With best regards,

Nanxing Yi
