# Supplementary material for: Baicalein Alleviates Osteoarthritis Progression in Mice by Protecting Subchondral Bone and Suppressing Chondrocyte Apoptosis Based on Network Pharmacology
Source: Front Pharmacol. 2022 Jan 10;12:788392. doi: 10.3389/fphar.2021.788392 (PMC8784526; doi:10.3389/fphar.2021.788392)
Supplement: Supplementary file 1 [file DataSheet1.ZIP › Additional files/Figure 4-8.pptx]

## Slide 1
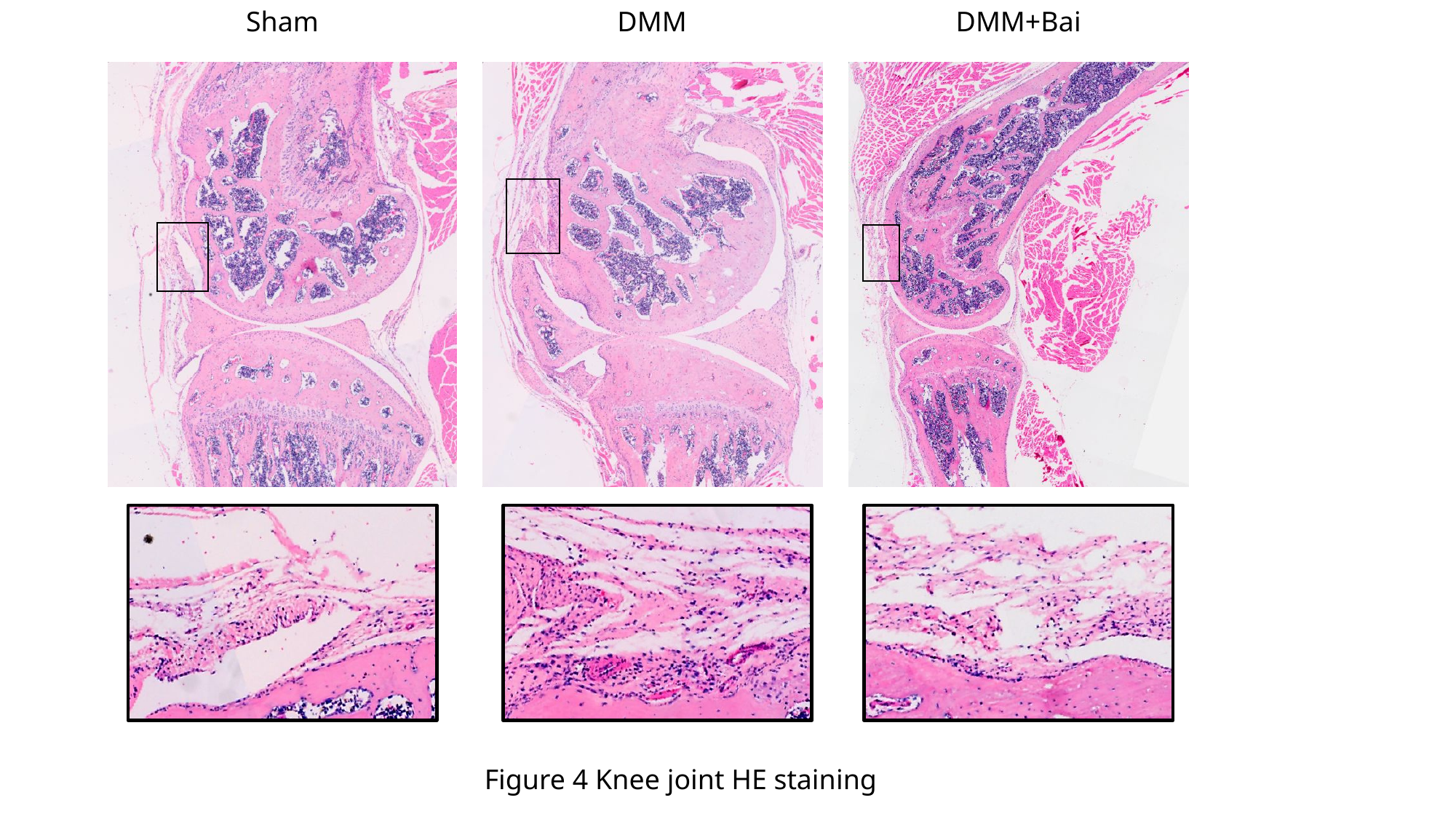

Sham
DMM
DMM+Bai
Figure 4 Knee joint HE staining

## Slide 2
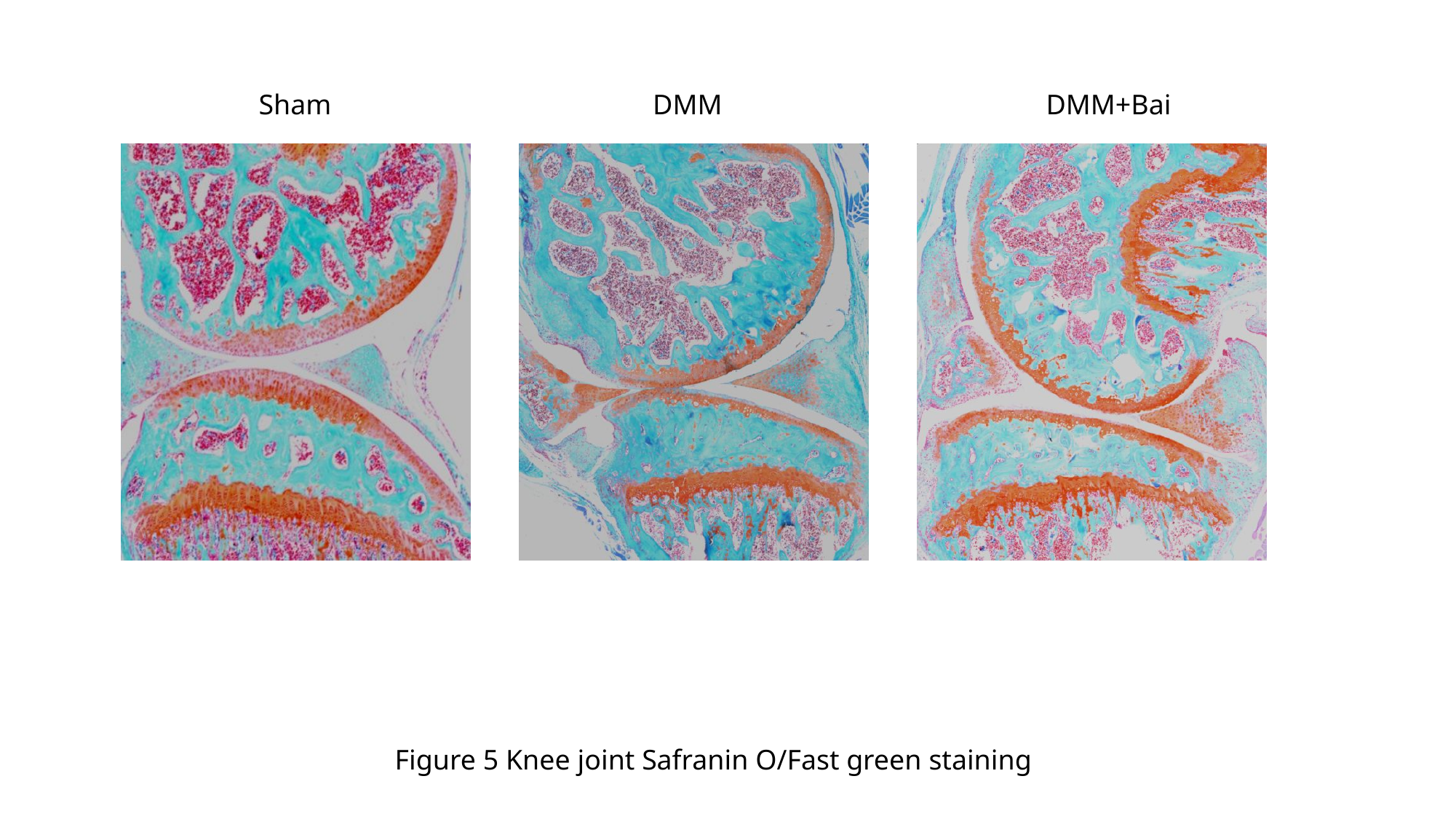

Sham
DMM
DMM+Bai
Figure 5 Knee joint Safranin O/Fast green staining

## Slide 3
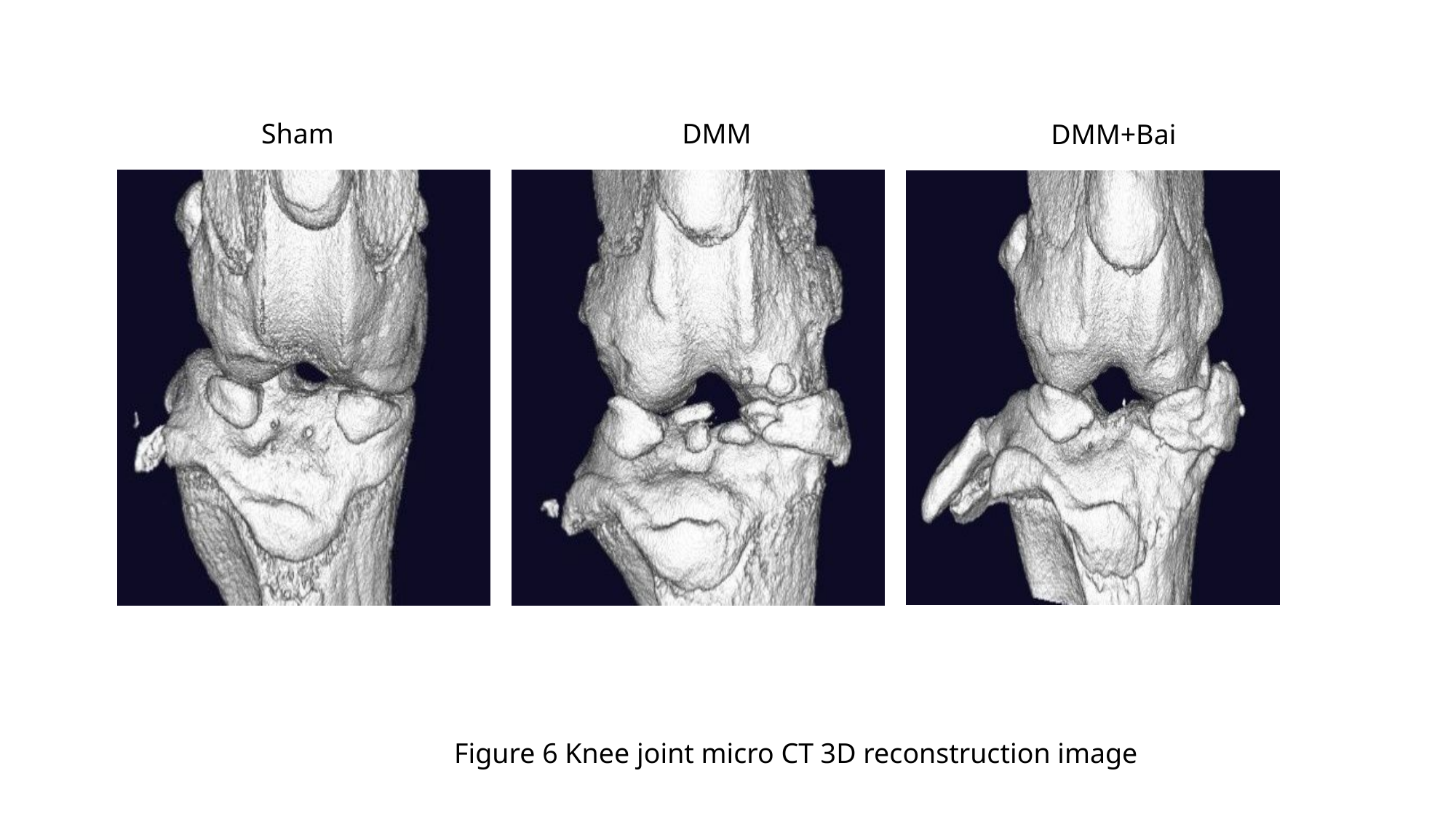

Sham
DMM
DMM+Bai
Figure 6 Knee joint micro CT 3D reconstruction image

## Slide 4
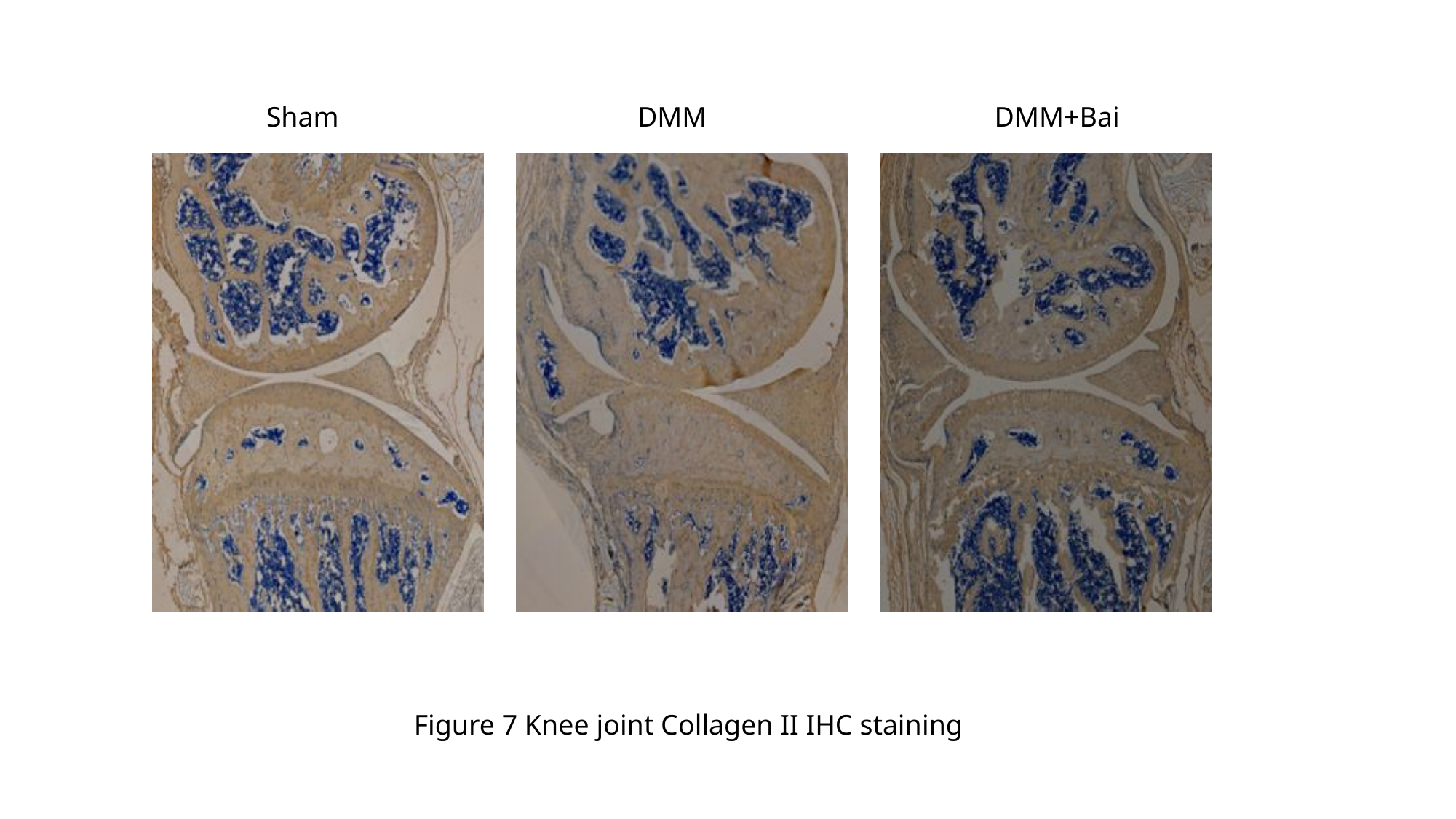

Sham
DMM
DMM+Bai
Figure 7 Knee joint Collagen II IHC staining

## Slide 5
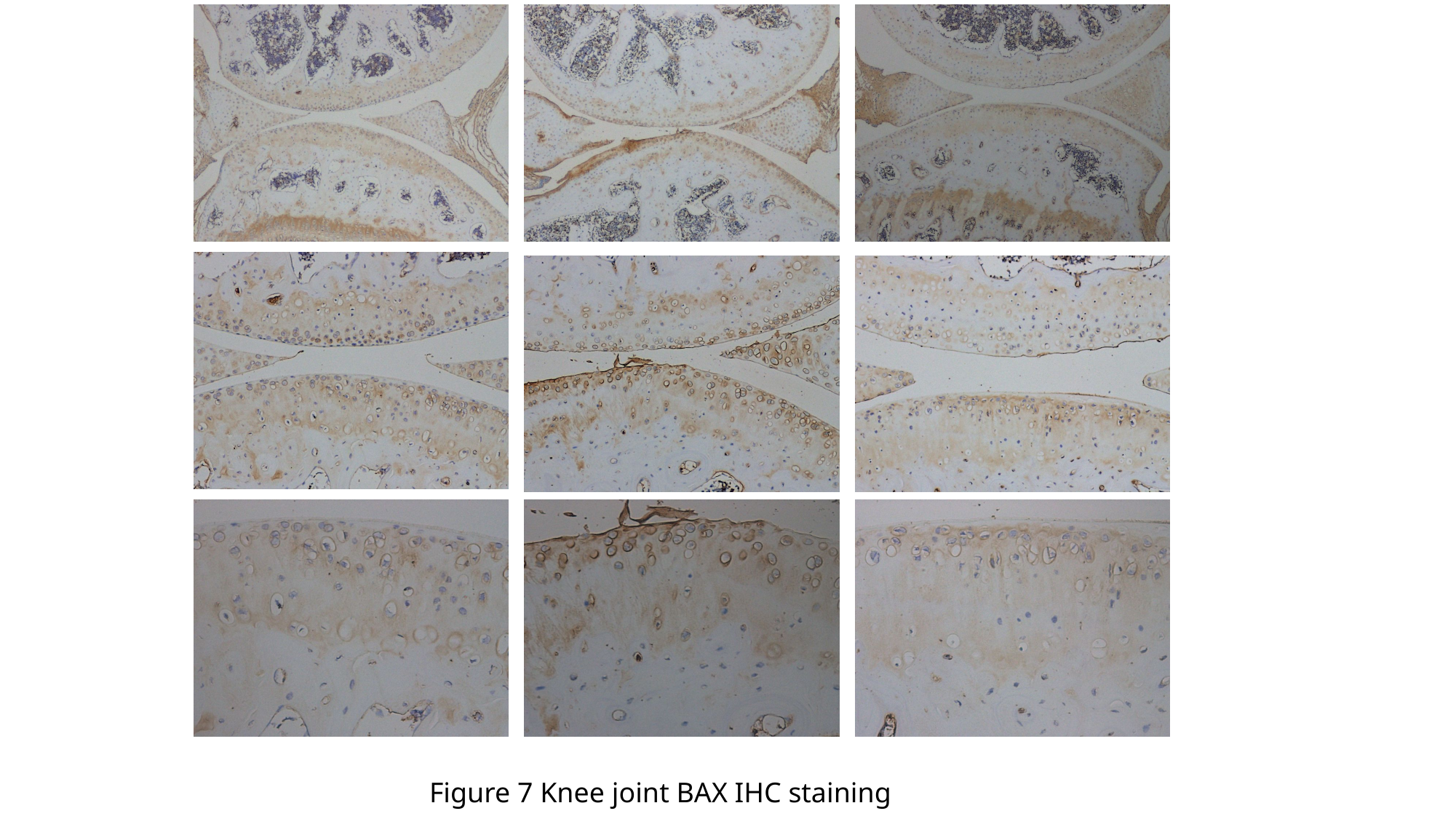

Sham
DMM
DMM+Bai
Figure 7 Knee joint BAX IHC staining

## Slide 6
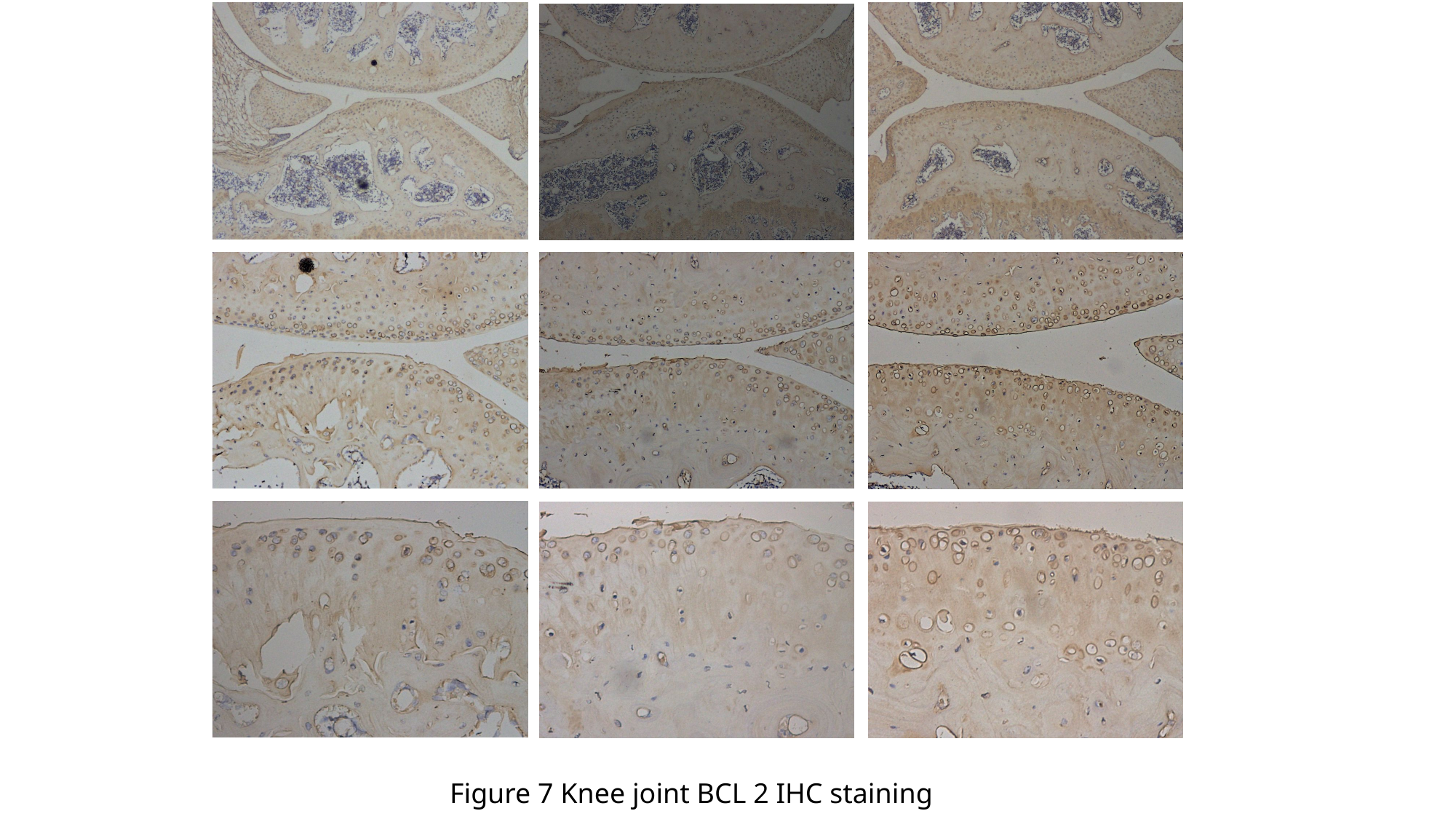

Sham
DMM
DMM+Bai
Figure 7 Knee joint BCL 2 IHC staining

## Slide 7
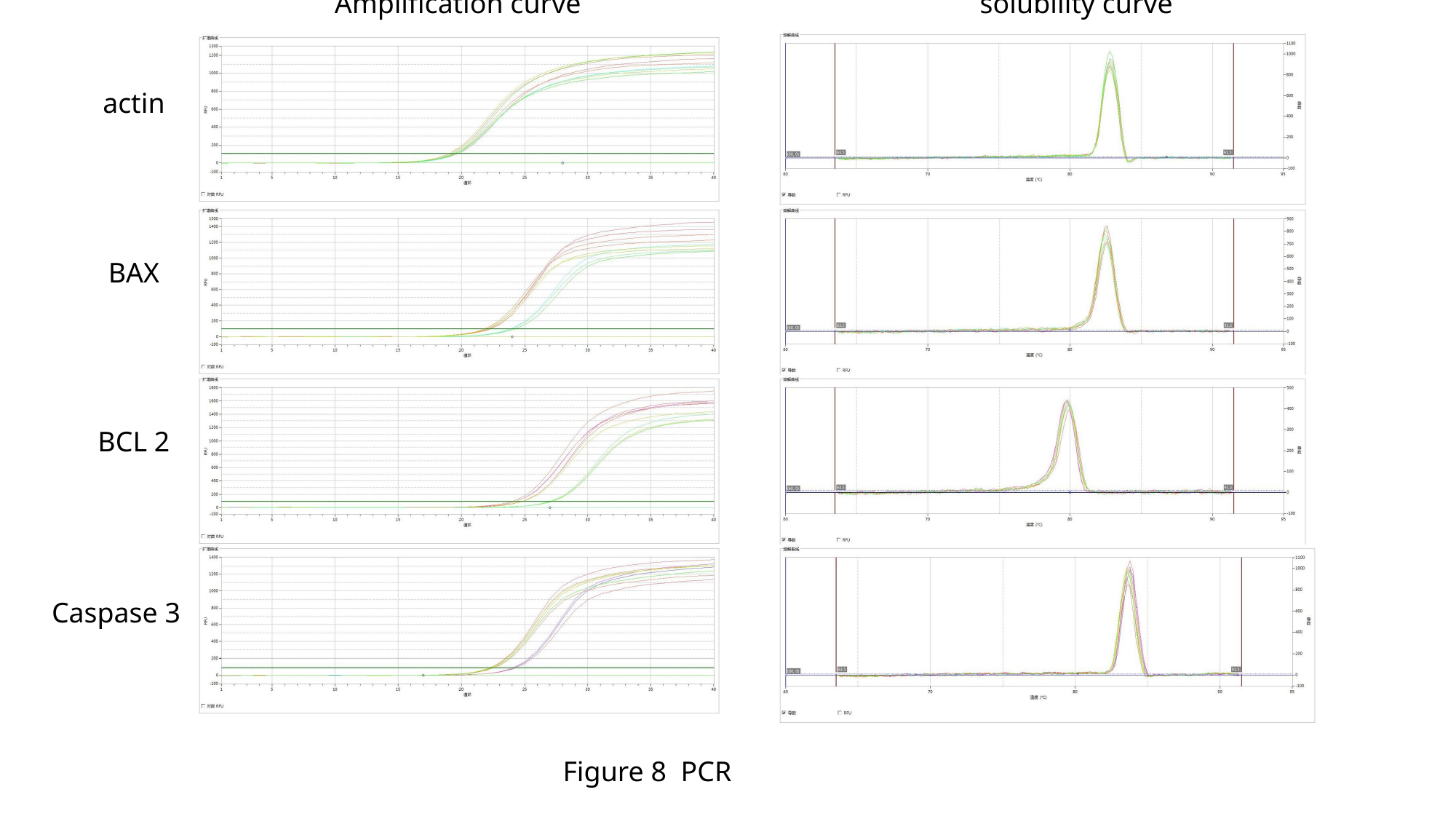

solubility curve
Amplification curve
actin
BAX
BCL 2
Caspase 3
Figure 8 PCR
